# Supplementary material for: Towards unified AI-driven fracture mechanics: the extended deep energy method (XDEM)
Source: Nat Commun. 2026 Aug 15;17:8492. doi: 10.1038/s41467-026-76748-1 (PMC13484471; doi:10.1038/s41467-026-76748-1)
Supplement: Supplementary file 3 [file 41467_2026_76748_MOESM3_ESM.zip › pic/Results/Stress_intensity_factor/Mixed/beta_75/crack_detailed_fields.pdf]

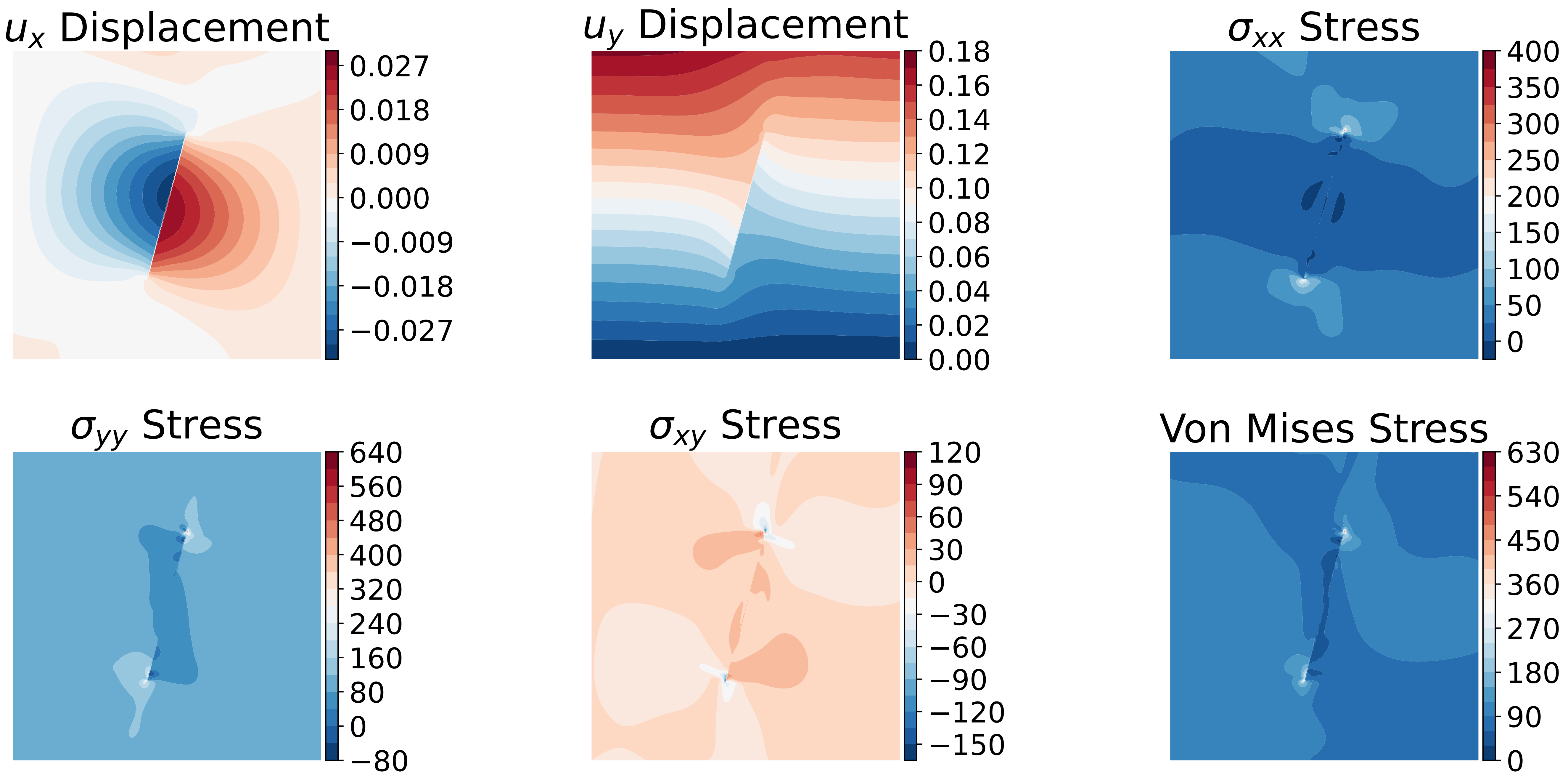
u_x Displacement
0.027
0.018
0.009
0.000
−0.009
−0.018
−0.027
u_y Displacement
0.18
0.16
0.14
0.12
0.10
0.08
0.06
0.04
0.02
0.00
σ_{xx} Stress
400
350
300
250
200
150
100
50
0
σ_{yy} Stress
640
560
480
400
320
240
160
80
0
−80
σ_{xy} Stress
120
90
60
30
0
−30
−60
−90
−120
−150
Von Mises Stress
630
540
450
360
270
180
90
0
